# Nanosize Titanium Dioxide Stimulates Reactive Oxygen Species in Brain Microglia and Damages Neurons *in Vitro*

**DOI:** 10.1289/ehp.10216

**Published:** 2007-08-03

**Authors:** Thomas C. Long, Julianne Tajuba, Preethi Sama, Navid Saleh, Carol Swartz, Joel Parker, Susan Hester, Gregory V. Lowry, Bellina Veronesi

**Affiliations:** 1 Department of Environmental Sciences and Engineering, University of North Carolina, Chapel Hill, North Carolina, USA; 2 Department of Civil and Environmental Engineering, Carnegie Mellon University, Pittsburgh, Pennsylvania, USA; 3 Constella Inc., Research Triangle Park, North Carolina, USA; 4 National Health and Environmental Effects Research Laboratory, U.S. Environmental Protection Agency, Research Triangle Park, North Carolina, USA

**Keywords:** BV2, environmental nanotoxicity, neurotoxicity, oxidative stress, P25, titanium dioxide

## Abstract

**Background:**

Titanium dioxide is a widely used nanomaterial whose photo-reactivity suggests that it could damage biological targets (e.g., brain) through oxidative stress (OS).

**Objectives:**

Brain cultures of immortalized mouse microglia (BV2), rat dopaminergic (DA) neurons (N27), and primary cultures of embryonic rat striatum, were exposed to Degussa P25, a commercially available TiO_2_ nanomaterial. Physical properties of P25 were measured under conditions that paralleled biological measures.

**Findings:**

P25 rapidly aggregated in physiological buffer (800–1,900 nm; 25°C) and exposure media (~ 330 nm; 37°C), and maintained a negative zeta potential in both buffer (–12.2 ± 1.6 mV) and media (–9.1 ± 1.2 mV). BV2 microglia exposed to P25 (2.5–120 ppm) responded with an immediate and prolonged release of reactive oxygen species (ROS). Hoechst nuclear stain was reduced after 24-hr (≥100 ppm) and 48-hr (≥2.5 ppm) exposure. Microarray analysis on P25-exposed BV2 microglia indicated up-regulation of inflammatory, apoptotic, and cell cycling pathways and down-regulation of energy metabolism. P25 (2.5–120 ppm) stimulated increases of intracellular ATP and caspase 3/7 activity in isolated N27 neurons (24–48 hr) but did not produce cytotoxicity after 72-hr exposure. Primary cultures of rat striatum exposed to P25 (5 ppm) showed a reduction of immunohistochemically stained neurons and microscopic evidence of neuronal apoptosis after 6-hr exposure. These findings indicate that P25 stimulates ROS in BV2 microglia and is nontoxic to isolated N27 neurons. However, P25 rapidly damages neurons at low concentrations in complex brain cultures, plausibly though microglial generated ROS.

The increased use of engineered nanoparticles in medical, agricultural, industrial, manufacturing, and military sectors Nanosize titanium dioxide is used in a variety of consumer products (e.g., toothpastes, sunscreens, cosmetics, food products) (Kaida et al. 2004), paints and surface coatings (Fisher and Egerton 2001) and in the environmental decontamination of air, soil, and water (Choi et al. 2006; Esterkin et al. 2005). Such widespread use and its potential entry though dermal, ingestion, and inhalation routes suggest that nanosize TiO_2_ could pose an exposure risk to humans, livestock, and eco-relevant species. Recent studies indicate that TiO_2_ is toxic to eco-relevant species (i.e., *Escherichia coli*, daphnia) (Adams et al. 2006) and mammals (Warheit et al. 2006, 2007). Numerous *in vitro* studies have reported OS-mediated toxicity in various cell types (Afaq et al. 1998; Beck-Speier et al. 2001; Gurr et al. 2005; Sayes et al. 2006; Wang et al. 2007b; Zhang and Sun 2004). However, the response of nerve cells to nanosize TiO_2_ has not been investigated *in vitro* or *in vivo*, except for a companion study (Long et al. 2006).

Because of their size and unusual properties, nanoparticles can enter the body and cross biological barriers relatively unimpeded. Several studies have reported that inhaled or injected nanosize particles enter systemic circulation and migrate to various organs and tissues (Kreyling et al. 2002; Takenaka et al. 2001) where they could accumulate and damage organ systems that are especially sensitive to oxidative stress (OS). The brain is one such organ, being highly vulnerable to OS because of its energy demands, low levels of endogenous scavengers (e.g., vitamin C, catalase, superoxide dismutase) and high cellular concentration of OS targets (i.e. , lipids, nucleic acids, and proteins). Recent experimental studies indicate that nanoparticles can cross the blood–brain barrier (Lockman et al. 2004) and enter (in low numbers) the central nervous system (CNS) of exposed animals (Kreyling et al. 2002; Oberdörster et al. 2004).

In the brain, OS damage is mediated by the microglia, a macrophage-like, phagocytic cell that is normally inactive unless confronted by potentially damaging xenobiotics. Their immediate and characteristic response (i.e., “oxidative burst”) to foreign stimuli involves cytoplasmic engulfment (i.e., phagocytosis), an increase in metabolic activity, and a change in cell shape, size and proliferation (Block et al. 2007). The NADPH-oxidase driven “oxidative burst” can be monitored by the immediate production and release of superoxide anions ( 
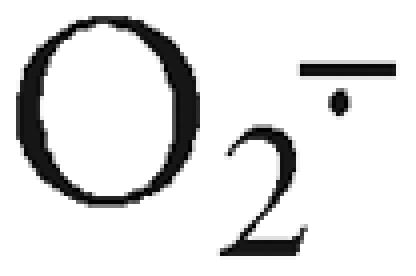
) that convert to multiple ROS such as hydrogen peroxide (H_2_O_2_), hydroxyl radicals, and peroxynitrites. The excess 
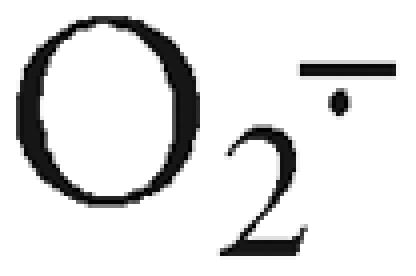
 arising from the oxidative burst can diffuse from the microglial plasma membrane and damage the proteins, lipids, and DNA of neighboring cells, especially neurons. Current thinking indicates that microglial-generated ROS underlie neurodegeneration (Block et al. 2007). Although the oxidative burst is the major source of ROS in the activated microglia, 
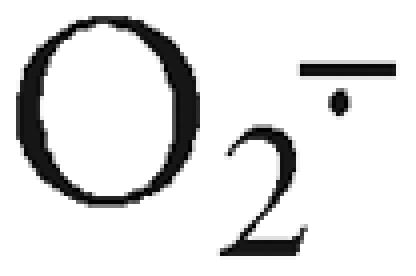
 is also generated as a by-product of normal mitochondrial energy production (i.e., bioenergetics). This results from the inefficient transfer of electrons along the electron transport chain (ETC) (Fariss et al. 2005). The levels of 
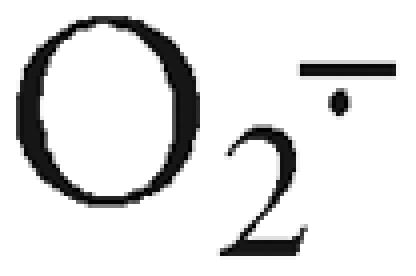
 generated from the ETC are relatively low and efficiently neutralized by matrix-located antioxidant enzyme systems (i.e., endogenous scavengers). However, the levels of ETC-generated 
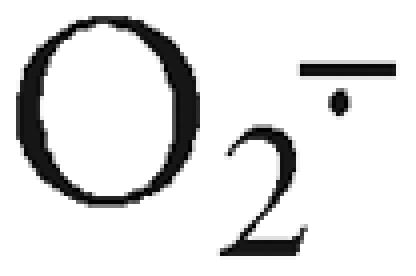
 can increase significantly if one or more of the enzymatic complexes in the ETC is inhibited.

To examine the possible neurotoxicity of TiO_2_, nerve cells critical to the pathophysiology of neurodegeneration (i.e., microglia, neurons) were exposed to a commercially available nanomaterial, Degussa P25. This material is an uncoated photo-active, largely anatase form of nanosize TiO_2_, not to be confused with the nonphotoactive nanomaterial currently used in sun blocks and cosmetics. P25 is a widely distributed material used for water treatment, self-cleaning windows, and antimicrobial coatings and paints. The BV2 microglia is an immortalized mouse cell line that responds to pharmaceutical agents, particulates, and environmental chemicals with characteristic signs of OS (Block et al. 2004; Wu et al. 2005). Its biochemical, morphological and genomic response to P25 exposure was examined in the present study. Because certain neuronal populations [such as dopaminergic (DA) neurons found in the brain striatum] are especially vulnerable to OS (Mattson 2001), the neurotoxicity of P25 was studied in the N27, an immortalized rat DA neuronal cell line (Zhou et al. 2000) and complex CNS cultures of embryonic rat striatum, which contains high numbers of DA neurons (Maier et al. 1994). Throughout the study, the physicochemical properties of P25 were described under exposure conditions that paralleled the biological response of these cells.

## Methods

### Physicochemical characterization

Commercial grade, uncoated nanosize Degussa P25 is a mixture of the anatase (70%) and rutile (30%) forms of TiO_2_. Anatase is the preferred form for use in catalysis because of its enhanced redox activity (Shi et al. 2007). Several physicochemical properties of nanosize particles such as zeta potential (i.e., surface charge) and particle aggregate size (Wiesner et al. 2006; Xia et al. 2006) have been associated with toxicity (Wiesner et al. 2006; Xia et al. 2006). A companion study measured the effect of P25 concentration on aggregate size in physiological buffer and culture media (Long et al. 2006). In the current study, the aggregate size and zeta potential of P25 at a median concentration (20 ppm) is studied under conditions (vehicle, time point, temperature) that parallel the biological response. Physicochemical properties of P25 (20 ppm) were measured in Hanks balanced salt solution (HBSS) at 25°C over a 120-min period to parallel the exposure parameters of ROS release in microglia. Measures were also taken in low serum (1%) culture media (RPMI 1640) at 37°C over 48 hr to parallel the neurotoxic response of N27 neurons. A Zeta Sizer Nano ZS (Malvern, Inc., Southborough, MA) was used to measure the hydrodynamic diameter (size) of P25 using the intensity-averaged distribution and the electrophoretic mobility of P25 was used to calculate its zeta potential using the Helmholtz-Smoluchowski equation.

### Cell culture

Immortalized mouse BV2 microglia and rat N27 mesencephalic neurons were grown, respectively, in Dulbecco’s modified Eagle’s medium (DMEM) or RPMI 1640 medium, supplemented with 10% fetal calf serum (FCS) and 1% penicillin–streptomycin (ATCC, Manassas, VA). Neurotoxicity studies used low (1%) serum RPMI 1640 exposure media. Tissue plugs of embryonic rat (Sprague-Dawley) brain striatum were purchased (BrainBits LLC, Springfield, IL; http://www.brainbitsllc.com) and upon receipt were triturated and plated on poly-D-lysine-coated 96-well plates (Nalge Nunc International, Rochester, NY) in Neurobasal/ B27 media (Invitrogen, Carlsbad, CA).

### Assays

Fluorescent and chemiluminescent probes were chosen to measure the changes resulting from the oxidative burst and interference with mitochondrial ETC (Invitrogen Corp. 2005). The immediate production of intracellular H_2_O_2_ generated from the oxidative burst was measured in BV2 microglia with Image-iT LIVE Green a dichlorodihydrofluorescein diacetate-based compound that reacts with intracellular esterases and fluoresces in the presence of ROS. The production of 
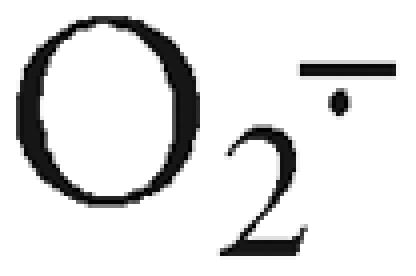
 resulting from interference with the mitochondria’s ETC was measured with MitoSOX Red. The viability and cytotoxicity of neuronal (N27) cultures were monitored using a lucerifase-based, chemiluminescence assay that measures intracellular levels of ATP (CellTiter-Glo; Promega, Inc., Madison, WI). Increases of caspase activity, an index of apoptotic entry was measured in both BV2 microglia and N27 neurons with Caspase-Glo 3/7 (Promega, Inc.). Loss of nuclear material (i.e., cytotoxicity) was measured with Hoechst 33342 (Invitrogen), a fluorescent probe that binds to adenine–thymine-rich regions of double-stranded nuclear DNA and indicates apoptotic loss of nuclear material (Oancea et al. 2006). All fluorescent probes were purchased from Molecular Probes (Eugene, OR) except for the chemiluminescent assays, CellTiter-Glo and Caspase-Glo 3/7.

For exposures, P25 (2.5–120 ppm) was ultrasonicated (~ 1 min) in 10 × stock concentrations in either HBSS or low serum exposure media. For ROS measurements, cells were exposed to the fluorescent probe (i.e., “loaded”) and washed with HBSS to remove any extracellular probe from the cell’s external environment.

### Genomics and bioinformatics

BV2 microglia were exposed (*n* = 3 wells/treatment) in 6-well plates to P25 (20 ppm) for 3 hr. Total RNA was extracted using TRIzol reagent (Invitrogen), purified, and its concentration determined using a NanoDrop ND-1000 Spectrophotometer (NanoDrop Technologies, Wilmington, DE). Large-scale gene analysis was performed by Expression Analysis (Durham, NC) using the Affymetrix Mouse Genome 430 2.0 GeneChip oligonucleotide array (Affymetrix, Santa Clara, CA) that measures approximately 39,000 transcripts. Target was prepared according to protocols outlined in the *Affymetrix Technical Manual* (Affymetrix Inc. 2004).

#### Data analysis

Affymetrix CEL files were analyzed using GC-robust multiarray (Wu et al. 2004) for array normalization and estimation of probe set intensities. Significance analysis of microarrays (SAM) (Tusher et al. 2001) was used to identify genes differentially expressed between P25-treated samples and the media control. Significantly different up-and down-regulated genes were analyzed by Ingenuity Pathway Analysis (IPA) software (Ingenuity Systems, Redwood City, CA; http://ingenuity.com/index.html) to determine *p*-values associated with Core canonical (metabolic and signaling) pathways and Tox Solution, which identifies relevant toxicity phenotypes and clinical pathology end points. Probesets that related to OS genes were analyzed separately using an IPA master list. In the graphic depiction of these analyses. The ratio of list genes to pathway genes is presented along with the Fisher exact test *p*-value. Pathways above a *p*-value threshold of 0.1 were discarded.

### Immunohistochemistry (IHC) and morphometry

Cultures were fixed for 30 min in 3.7% paraformaldehyde, blocked with a mixture of 1% BSA, 0.4% Triton X-100, and 4% normal horse serum (20 min at room temperature, RT), and incubated in a 1:200 dilution of monoclonal mouse anti-human, neuron-specific enolase (NSE) for 30 min at RT (Dako Inc., Ft. Collins, CO). Visualizaton with streptavidin followed protocol of the LSAB 2 System-HP kit from Dako. IHC stained striatal cultures were analyzed morphometrically for neuronal loss. Six (10 ×) photographs of each well (*n* = 3/treatment) were taken using a Nikon TE300 inverted microscope and a cooled-frame CCD camera (Orca I; Hamamatsu Photonics, Hamamatsu City, Japan). Each digitized image was analyzed using MetaMorph 7.0 software (Molecular Devices, Sunnyvale, CA). Populations of control, NSE-stained neurons were “binned” according to size and shape parameters using the integrated morphometric analysis mode. The total area of NSE-stained figures (cell bodies with attached axons) that fell within these parameters was calculated and compared with cultures treated with P25 (5 ppm; 6–48 hr). Data were collected in Excel 2003 (Microsoft Corp., Redmond, WA) and transferred to GraphPad Prism 5 for graphing of the histogram (Graphpad Software, Inc., San Diego, CA; www.graphpad.com).

### Light (LM) and transmission electron microscopy (TEM)

For TEM examination, cells were exposed in 6-well plates to P25 particles (20 ppm) for 3 hr. After exposure, cells were washed in warm HBSS to remove all noninternalized particles and fixed overnight in cold 2.5% cacodylate-buffered glutaraldehyde (Poly Scientific, Bayside NY). Cells were processed for TEM using standard procedures (Phillips 1998) and examined with a Zeiss LEO electron microscope (Carl Zeiss SMT Inc., Peabody, MA). LM preparations were examined as toluidine blue stained 1-μm epoxy sections or in unstained glutaraldehyde-fixed samples. Both types of LM samples were photographed with a Nikon TE300 inverted microscope.

### Statistics

Spectrophotometric data were collected using SoftMax Pro 4.8 software (Molecular Devices). Graphing and statistics were done using Excel 2003 or GraphPad Prism 5. The mean response value (*n* = 6) of each concentration treatment was calculated. Data from several time intervals were normalized to show a time-course response. Data were analyzed using a one-way analysis of variance (ANOVA) with Dunnett’s test to determine significance (*p < 0.05) relative to its unexposed control.

## Results

### BV2 (ROS)

**Figure 1:**
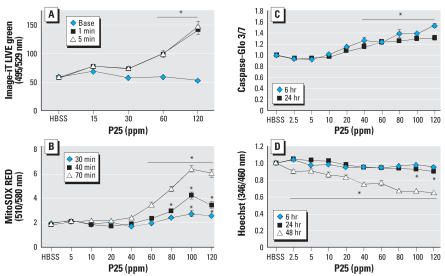
(*A*) The immediate production of intracellular H_2_O_2_ generated from the oxidative burst was measured in BV2 microglia with Image-iT LIVE Green. Cells were incubated (30 min, 37°C) in 25 μM Image-iT LIVE Green and exposed to P25. Significant increases of fluorescence first occurred in response to P25 (60 ppm; 1 min). (*B*) The production of 
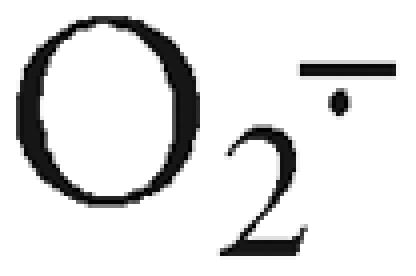
 resulting from interference with the mitochondria’s ETC was measured with MitoSOX Red. BV2 microglia, incubated in 2 μM (10 min, 37°C) showed a delayed but significant increase in fluorescence after 30-min exposure to ≥100 ppm P25. (*C*) Significant increases of caspase 3/7 activity were first seen by 6 hr in response to ≥40 ppm P25 and remained at this level for 24 hr. (*D*) Apoptotic loss of nuclear material, as measured with Hoechst stain, was first noted after 24 hr in response to P25 (≥100 ppm) and involved all concentrations by 48 hr.

**Figure 2:**
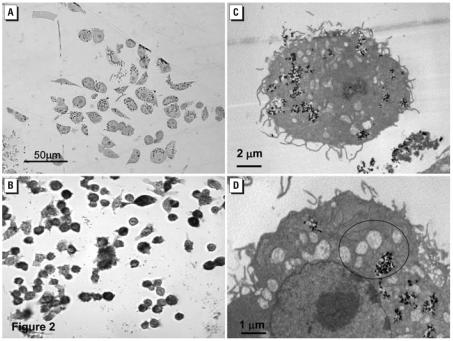
BV2 microglia exposed to P25 (20 ppm) were examined with both LM and TEM. (*A*) The toluidine blue stained cytoplasm of BV2 microglia housed numerous, light-refractive P25 aggregates after 3-hr exposure. (*B*) LM examination of unstained, fixed cells exposed to P25 for 48 hr indicated that the cellular membranes were fragmented and showed granular cytoplasm and centralized nuclei. Magnification × 1,200. (*C*) TEM examination of the P25 exposed BV2 microglia indicate phagocytic internalization of the P25 aggregates after 3 hr. (*D*) Higher magnification of the BV2 microglial cytoplasm indicated swollen and disrupted mitochondria (circles) in proximity to the P25 aggregates.

Measures of H_2_O_2_ released from both the oxidative burst and inhibition of the ETC were collected. BV2 microglia responded to P25 at ≥60 ppm with a rapid (1–5 min) release of H_2_O_2_ as measured with Image-iT LIVE Green (Figure 1A). Significant release of 
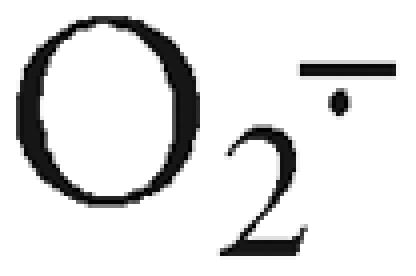
 as measured by MitoSOX Red first occurred at 30 min in response to ≥100 ppm P25 and only responded to concentrations ≥60 ppm after 70-min exposure (Figure 1B). Significant increases in caspase 3/7 activity, which signal the cell’s entry into apoptosis (Fariss et al. 2005), were first measured at 6 hr in response to ≥40 ppm P25 and remained at this level by 24 hr (Figure 1C). BV2 cells showed reduced nuclear staining in response to ≥100 ppm P25 after 24 hr and ≥2.5 ppm P25 after 48-hr exposure as measured by Hoechst dye (Figure 1D). LM examination of P25 (20 ppm) exposed BV2 microglia indicated that they internalized aggregates of P25 in small clusters by 3-hr exposure (Figure 2A) and by 48 hr responded with fragmented cellular membranes and numerous condensed nuclear figures, suggestive of apoptosis (Figure 2B). TEM examination of the BV2 microglia indicated phagocytic internalization of the P25 (20 ppm, 3 hr) (Figure 2C) and swollen, disrupted microglia in the vicinity of these aggregates (Figure 2D).

### BV2 genomics and bioinformatics

**Figure 3:**
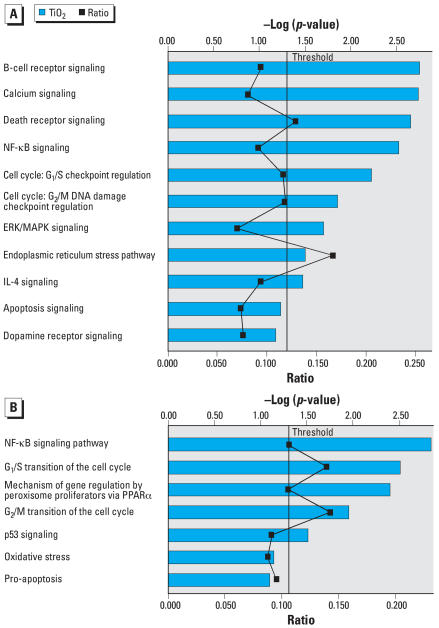
BV2 microglia were exposed to P25 (20 ppm) for 3 hr and prepared for microarray analysis. IL-4, interleukin 4; PPARα, peroxisome proliferator-activated receptor α (*A*) IPA’s Core analysis (metabolic/signaling pathways) indicated that up-regulated genes were clustered around signaling pathways involved with apoptosis, Death receptor families (i.e., caspase activation), calcium signaling, inflammation (NF-κB), and cell cycling and maintenance. (*B*) Toxicity Pathway analysis indicated that P25 up-regulated pathways were primarily associated with inflammatory (NF-κB), cell cycling and pro-apoptotic activities.

**Figure 4:**
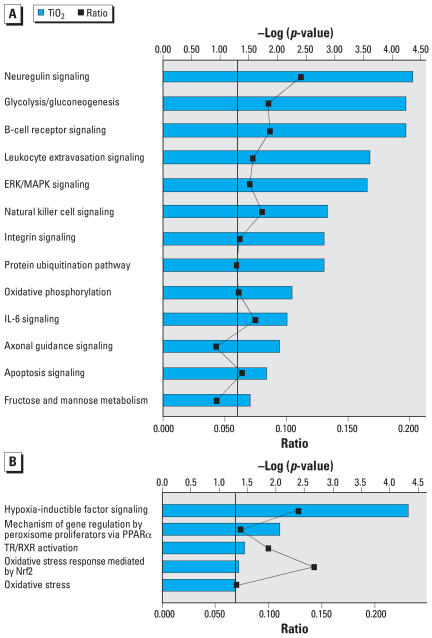
BV2 microglia were exposed to P25 (20 ppm) for 3 hr. IL-6, interleukin 6, PPARα, peroxisome proliferator-activated receptor α TR/RXR, thyroid hormone receptor/retinoid X receptor. (*A*) Core analysis of the down-regulated genes showed clustering around pathways associated with adaptive change and key energy production pathways. (*B*) Toxicity Pathway analysis indicated that P25 down-regulated genes in pathways associated with hypoxia, peroxisomes, and Nrf2-mediated oxidative stress.

**Figure 5:**
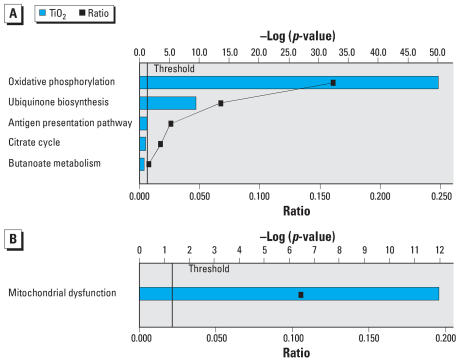
BV2 microglia were exposed to P25 (20 ppm) for 3 hr. (*A*) Canonical analysis of all P25 affected genes associated with OS indicated that they largely clustered around key energy pathways involving oxidative phosphorylation, biosynthesis of ubiquinone (involved in shuttling electrons in the ETC) and the citric acid cycle. (*B*) Toxicity Pathway analysis localized these pathways further to mitochondrial dysfunction.

SAM identified 1,755 probesets that were differentially and significantly expressed (1,195 up-regulated, 560 down-regulated) in P25-treated cultures relative to medium controls. Graphs depicting this analysis are shown in Figures 3–5. In each graph, terms describing the various IPA Core and Toxicity Pathways are shown on the left x-axis. The top y-axis displays the –log_2_ of the *p*-value and is depicted as a straight red line. The *p*-value indicates how likely P25-affected genes are associated with IPA genes associates with that particular function or pathway. The height of the blue bar indicates its significance to that gene function or pathway. The wavy line on the lowery-axis represents the ratio or percent of P25-affected genes relative to a particular IPA pathway.

Core canonical analysis (Figure 3A) indicated that P25 up-regulated genes were clustered around signaling pathways involved with B-cell receptor (gene transcription in the immune response), the Death receptor (tumor necrosis factor receptor family; apoptotic initiating pathways; caspase activation), apoptosis, calcium, and inflammation [nuclear factor (NF)-κ B]. Several up-regulated cell cycling and maintenance pathways included neuregulin and ERK/MAPK (extra-cellular signal-regulated kinase/mitogen-activated protein kinase) receptor (growth factors for cell proliferation, differentiation, migration, survival, and fate). Toxicity analysis (Figure 3B) indicated a strong pathway association with pathways associated with inflammation (NF-κ B), cell cycling, oxidative stress (peroxisomes) and pro-apoptotic activities. P25’s down-regulated genes (Figure 4A) were associated with adaptive change (e.g., B-cell receptor, ERK/MAPK) and energy production (glycolysis, gluconeogenesis, oxidative phosphorylation). Toxicity analysis indicated that down-regulated genes were associated with pathways triggered by response to low oxygen availability (i.e., hypoxia-inducible factor), peroxisomes, and Nrf2-mediated OS (Figure 4B). Canonical analysis of the P25 affected genes associated with OS indicated that the majority clustered around key energy pathways involving oxidative phosphorylation, biosynthesis of ubiquinone (involved in shuttling electrons in the ETC) and the citric acid cycle (Figure 5A). Toxicity Pathway analysis indicated that these pathways were associated with mitochondrial dysfunction (Figure 5B).

### Neurotoxicity

**Figure 6:**
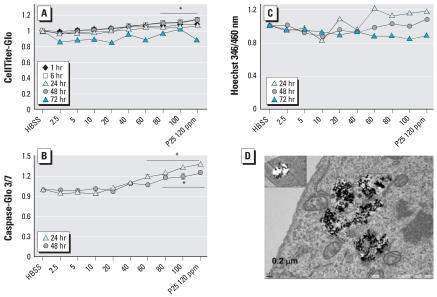
(*A*) N27 neurons were exposed (1–72 hr) to P25 (2.5–120 ppm) and intracellular ATP levels measured with CellTiter-Glo. Significant increases were seen as early as 1 hr postexposure to ≥80 ppm and continued until 48 hr in response to ≥40 ppm. (*B*) Significant increases (*p* < 0.05) in caspase 3/7 activity were first seen in N27 neurons after 24 hr in response to ≥40 ppm P25. (*C*) Significant reductions of Hoechst stain did not occur in response to P25 (2.5–120 ppm) at any time point. (*D*) TEM of P25 (20 ppm, 3 hr) treated N27 neurons showed numerous membrane-bound aggregates. An amorphous substance was seen within the vacuoles (insert). In addition, individual nanosize P25 particles (circle) were noted throughout the cytoplasm. Mitochondria in nearby proximity showed no evidence of disruption or swelling.

The direct (in the absence of microglia) neurotoxicity of P25 and that mediated by microglia-generated ROS was addressed, respectively, in isolated rat DA neurons (N27) and in primary cultures of rat striatum. P25 increased intracellular levels of ATP in N27 beginning at 1 hr (≥80 ppm) and continued over 48 hr (≥40 ppm) (Figure 6A). Caspase 3/7 activity, an indicator of apoptosis, significantly increased at both 24- and 48-hr exposure (≥40 ppm) (Figure 6B). Apoptotic damage to isolated N27 neurons, using Hoechst 33342 nuclear stain, was not seen even after a 72-hr exposure to P25 (2.5–120 ppm) (Figure 6C).

Ultrastructurally, both nanosized and large aggregates of P25 were seen in the N27 cytoplasm after 3-hr exposure to P25 (20 ppm). P25 aggregates were randomly located thoughout the neuronal cytoplasm (Figure 6D) and appeared to be encased in membrane-bound vacuoles (Figure 6D, inset). No evidence of phagocytosis or pinocytosis (i.e., elaboration of pseudopodia) was observed, suggesting that the particles impacted the cell body by sedimentation. In contrast to the disrupted organelles noted above in BV2 microglia, mitochondria appeared ultrastructurally normal in the N27 neurons, in spite of their close proximity to P25 aggregates.

**Figure 7:**
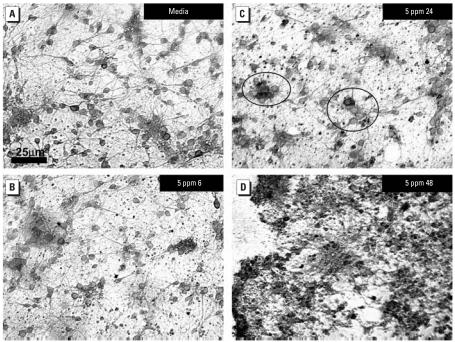
LM histology of IHC rat embryonic striatum. Confluent cultures of embryonic were exposed to 5 ppm P25 for 6–48 hr, IHC stained with NSE and morphometrically analyzed. (*A*) Untreated cultures consisted of a dense plexus of neurons and glia. (*B*) Axonal beading and cellular granularity were seen as early as 6 hr postexposure. (*C*) Evidence of apoptosis (circles) was documented by 24 hr. (*D*) Complete disruption and loss of cellular integrity was noted by 48 hr postexposure to 5 ppm P25.

**Figure 8:**
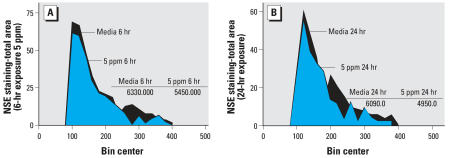
Morphometric analysis was conducted on NSE-stained cultures of mouse striatum. These data indicated that the total area of NSE-stained neurons was reduced by 14% after 6-hr exposure (*A*) and 19% after 24 hr (*B*) to P25 (5 ppm).

IHC stained cultures of rat brain striatum, exposed to P25 (5 ppm) were photographed (Figure 7A–D) and analyzed morphometrically. Results indicated that the total area of NSE-stained neurons were reduced by 14% after 6-hr exposure and 19% after 24-hr exposure (Figure 8A,B).

### Physicochemistry

**Figure 9:**
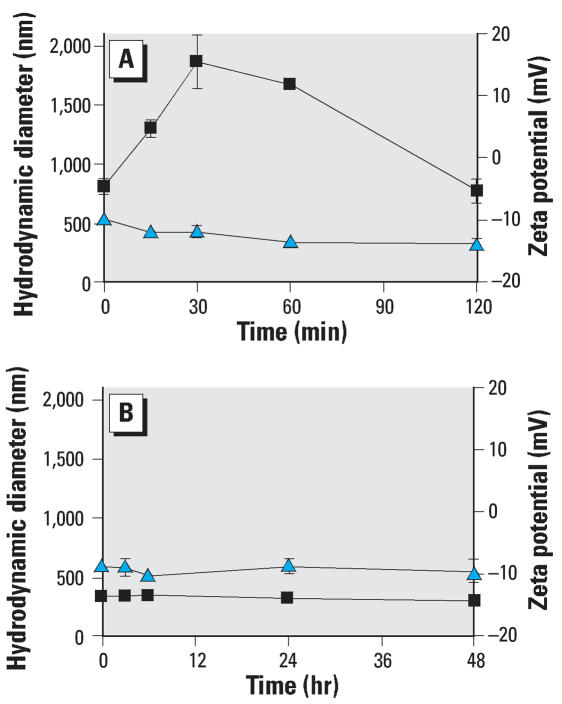
The aggregate size and zeta potential of P25 (20 ppm) were measured in both HBSS and RPMI at conditions that paralleled the biological responses. (*A*) P25 aggregates reached 1,900 nm in size over a 30-min measurement in HBSS (25°C) and maintained > 1,000 nm size for the 2-hr exposure period. The zeta potential of P25 (blue triangles) initially measured –9.8 mV and decreased slightly over the 2-hr period. (*B*) Both the aggregate size (black squares; 300–350 nm) and zeta potential (blue triangles) (–8 mV to –10 mV) of P25 remained stable when measured in RPMI over the 48-hr period.

The aggregate size and zeta potential of P25 (20 ppm) were measured in relevant exposure vehicles (HBSS, RPMI) at time points that paralleled the biological response (Figure 9A,B). In HBSS, the hydrodynamic diameter of P25 aggregates ranged from 800 to 1,900 nm (30 min) and decreased to 770 nm (2 hr) as the larger aggregates settled from solution. The zeta potential of P25 (20 ppm) in HBSS (pH 7.6) ranged from –9.78 to –13.8 mV after 2 hr (25°C) (Figure 9A). In low-serum RPMI exposure medium, P25 quickly aggregated but remained relatively stable in suspension (300–350 nm) over the 48-hr exposure period. The zeta potential ranged from –8.54 to –10.1 mV over 0–48 hr (37°C) in low-serum RPMI media (Figure 9B).

## Discussion

The present data indicate that Degussa P25 stimulates BV2 microglia to release ROS and affects genomic pathways associated with cell cycling, inflammation, apoptosis and mitochondrial bioenergetics. Although, several adaptive pathways (neuregulin ERK/MAP kinase) (Pagès et al. 1999) were differentially affected, the ubiquinone, biosynthetic pathway which functions as an electron carrier in the mitochondrial ETC and also acts as antioxidant (Artuch et al. 1999), and mitochondrial bioenergetic pathways involving oxidative phosphorylation, glycolysis, etc. were severely depressed which would create levels of ROS and ultimately OS in the cell (Fariss et al. 2005). P25 appeared to be non-toxic to isolated DA neurons (N27) even after 72 hr. However, when examined in primary cultures of brain striatum which contain microglia, neuronal loss occurred by 6 hr in response to only 5 ppm. This shift in dose-response, coupled with cellular and genomic evidence of P25’s effect on inflammatory and apoptotic pathways and disruption of energy pathways in BV2 microglia, suggest that the potent neurotoxicity of P25 seen in complex cultures was mediated though microglia-generated ROS. The microglia’s release of H_2_O_2_ from the oxidative burst and ETC, if excessive, can activate caspase 8 and its downstream effectors caspase 3/7, inducing apoptosis though extrinsic cell death pathways (De Giorgi et al. 2002). Stimulation of mitochondrial apoptotic pathways (e.g., caspase 3/7) was noted biochemically and genomically in BV2 microglia, and apoptotic morphology was shown in both isolated BV2 microglia and in cultures of striatum. These data indicate that OS-mediated apoptosis played a signature role in P25 neurotoxicity.

Ultrastructurally, the phagocytosis of P25 aggregates by BV2 microglia and the strong association of mitochondrial disruption with these aggregates have been previously reported (Long et al. 2006). Fractal aggregates can maintain the large surface area, sharp crystallite edges, and other characteristics of individual nanoparticles (Phenrat et al. 2007). Membrane-bound P25 aggregates were also seen within the N27 cytoplasm. However, no morphological evidence of phagocytosis, pinocytotosis or endocytosis was noted. Because of this, the possibility that P25 aggregates sedimented from the exposure medium onto the cells and became incorporated into cytoplasmic lysosomes cannot be excluded. Nanosize particles were also documented lying free in the neuronal cytoplasm. The manner by which such nanoparticles particles enter the cell cytoplasm is still a matter of discussion and is thought to involve mechanisms distinct from phagocytosis and endocytosis (Rothen-Rutishauser et al. 2006).

The biological interactions of nanoparticles are associated with physical properties such as surface area, particle shape, zeta potential, and aggregate size (Wiesner et al. 2006). For valid interpretation of nanotoxicity data, these properties must be determined under conditions that parallel the biological exposures. Our data indicated that the exposure conditions (i.e., vehicle, temperature) significantly modified P25’s particle size and zeta potential which could affect its interaction with biological systems and its ultimate toxicity. Particle (or aggregate) size determines if a particle enters the cellular environment though ROS-producing phagocytosis, through endocytosis, or some undefined mechanisms (Champion and Mitragotri 2006; Sabokbar et al. 2003). The surface charge or zeta potential of a particle affects its aggregation in solution and its behavior in an electric or ionic field. The surface charge of a particle also determines its interactions with specific biological receptors. Polymodal receptors located in the cellular membrane of microglia and macrophages (e.g., TRPV1, Mac-1) are sensitive to protons (i.e., charge) or repeating patterns of charge (Block et al. 2007; Husemann et al. 2002; Reilly et al. 2006) like those found on crystalline metal oxide nanoparticles. The activation of these receptors triggers various signal transduction pathways that determine the cell’s ultimate fate. Scavenger receptors have been implicated in mediating the cytotoxicity of alveolar macrophages exposed to TiO_2_ (Kim et al. 1999). Studies have also shown that TRPV1 receptors located on rat primary microglia stimulate OS-mediated apoptotic cell death (Kim et al. 2005). The role of these receptors in mediating P25 apoptosis in BV2 microglia is currently being examined using pharmacological and electrophysiological endpoints.

In summary, this study describes the *in vitro* neurotoxicity of a widely used nano-material, P25. This material appears to be non-toxic to isolated N27 neurons but stimulates BV2 microglia to produce ROS and damages OS-sensitive neurons in cultures of brain striatum.

## Correction

Authorship of this article has been modified from the orignal article published online.
